# Comparison of all-cause mortality with different blood glucose control strategies in patients with diabetes in the ICU: a network meta-analysis of randomized controlled trials

**DOI:** 10.1186/s13613-025-01471-x

**Published:** 2025-04-09

**Authors:** Xi Li, Jiahao Meng, Xingui Dai, Pan Liu, Yumei Wu, Shuhao Wang, Heng Yin, Shuguang Gao

**Affiliations:** 1 Department of Orthopaedics, Xiangya Hospital, Central South University, Changsha, 410008 Hunan China; 2 Department of Critical Care Medicine, Affiliated Chenzhou Hospital (The first People’s Hospital of Chenzhou), University of South China, Chenzhou, China; 3 Key Laboratory of Aging-related Bone and Joint Diseases Prevention and Treatment, Xiangya Hospital, Ministry of Education, Central South University, Changsha, China; 4 National Clinical Research Center of Geriatric Disorders, Xiangya Hospital, Central South University, Changsha, Hunan China

**Keywords:** Intensive care unit, Diabetes, Glucose control, All-cause mortality

## Abstract

**Background:**

The optimal glucose control strategy for intensive care unit (ICU) patients with diabetes remains a topic of debate. This study aimed to compare the effects of strict glucose control, intermediate strict glucose control, liberal glucose control, and very liberal glucose control on reducing all-cause mortality in ICU patients with diabetes through a network meta-analysis.

**Methods:**

We conducted a search in PubMed, Cochrane Library, Embase, and Web of Science for randomized controlled trials comparing different glucose control strategies in ICU patients with diabetes up to October 1, 2024. The primary outcome was all-cause 90-day mortality. The Risk of Bias 2 tool was used to assess bias in the included studies. Data analysis was performed using Stata (version 17).

**Results:**

A total of 12 randomized controlled trials involving 5,297 participants were included in the final analysis. The results showed that there was no statistically significant difference between the four glucose control strategies in reducing all-cause 90-day mortality. The surface under the cumulative ranking (SUCRA), which was used to rank the strategies and display the probability of each strategy being ranked first, showed the following: intermediate strict control (SUCRA 88%), liberal control (SUCRA 55.3%), very liberal control (SUCRA 40.3%), and strict control (SUCRA 16.5%). The cumulative probability of each strategy’s rank in reducing all-cause mortality, from best to worst, showed that the most likely ranking was intermediate strict control, liberal control, very liberal control, and strict control.

**Conclusions:**

In ICU patients with diabetes, no significant statistical difference was observed among the four glucose control strategies in reducing all-cause 90-day mortality. The SUCRA rankings are hypothesis-generating and require further validation. Therefore, the current evidence is insufficient to definitively conclude that any one strategy is superior to the others in reducing mortality.

**Supplementary Information:**

The online version contains supplementary material available at 10.1186/s13613-025-01471-x.

## Introduction

Diabetes is a chronic metabolic disorder characterized by elevated blood glucose levels, and it is relatively common among patients in the intensive care unit (ICU) [1, 2]. ICU patients with diabetes face a higher risk of complications, including prolonged hospital stays, increased mortality, and poorer recovery outcomes [3]. The presence of diabetes complicates the management of critically ill patients, as stress-induced hyperglycemia is often observed in these patients, further worsening their prognosis [3, 4]. This is particularly true for patients with undiagnosed diabetes or poorly controlled pre-existing diabetes, as stress-induced hyperglycemia is associated with worse outcomes, including higher infection rates, organ dysfunction, and extended ICU stays [5, 6].

Recent studies have emphasized the importance of blood glucose management in ICU patients with diabetes to mitigate these risks [3, 7]. However, the optimal glucose control strategy for critically ill patients remains controversial [5]. Intensive glucose control (i.e., maintaining lower blood glucose levels) has been proposed to reduce morbidity and mortality, but concerns over the risk of hypoglycemia have sparked debates about its safety and effectiveness [5, 6]. On the other hand, more relaxed glucose control strategies, which aim to avoid hypoglycemia, may carry a lower risk but have also been associated with worse outcomes in some settings [5, 6].

Recently, a traditional meta-analysis by Defante et al. [8] showed that in ICU patients with diabetes, there was no statistically significant difference in all-cause mortality between strict blood glucose control and liberal blood glucose control. However, many studies have also compared intermediate strict glucose control and liberal glucose control, which have not yet been included in relevant meta-analyses [9–13].

Thus, we conducted the first network meta-analysis to comprehensively compare the effects of strict glucose control, intermediate strict glucose control, liberal glucose control and very liberal glucose control on all-cause mortality in ICU patients with diabetes.

## Methods

This study was conducted in accordance with the 2020 PRISMA (Preferred Reporting Items for Systematic Reviews and Meta-Analyses) guidelines [14]. After conducting a preliminary analysis to determine the feasibility of the topic, we registered the protocol on PROSPERO (CRD42024629407).

### Search strategy and selection criteria

A comprehensive literature search was conducted on October 1, 2024, across several databases, including PubMed, Cochrane Library, Embase, and Web of Science, using both MeSH terms and broad keywords such as “glucose,” “strict,” “intensive,” “tight,” “loose,” “diabetes,” “intensive care unit,” and “critically ill.” The detailed search strategy was uploaded in the attachment (Supplement, Search strategy). Additionally, we manually searched the reference lists of relevant review articles. After completing the initial search, we repeated the process to include the most recent studies.

The retrieved literature was imported into EndNote X9. After removing duplicate references, two reviewers independently screened the studies based on titles and abstracts. Full-text articles of potentially eligible studies were then assessed according to the inclusion and exclusion criteria. Based on the PICO framework, the inclusion criteria were: ICU patients with diabetes, interventions involving different glucose control strategies (strict glucose control, intermediate strict control, liberal control and very liberal control), and the primary outcome of all-cause 90-day mortality. Exclusion criteria included non-randomized controlled trials and studies for which the full text could not be retrieved. Any discrepancies were resolved through discussion with a senior author.

### Data extraction

The two reviewers independently extracted key data from the selected studies using a standardized data collection form. This form captured essential details, including the first author, year of publication, country, sample size, target glucose levels for each group, type of diabetes, definition of hypoglycemia, follow-up duration, and primary outcomes. If any data were missing, efforts were made to contact the corresponding authors for clarification. The primary outcome of interest was all-cause 90-day mortality. Any discrepancies were resolved through discussion between the reviewers.

### Risk of bias assessment

The potential risk of bias in the randomized controlled trials (RCTs) was evaluated using the Rob2 tool, a revised assessment method that focuses on five core domains [15]: the randomization process, deviations from the intended interventions, missing outcome data, outcome measurement, and selection of the reported result. Each domain was carefully examined for possible bias and rated as low risk, some concerns, or high risk based on the available evidence and specific study circumstances. The overall bias for each study was then determined by synthesizing the evaluations across all five domains, leading to a classification of low risk, some concerns, or high risk.

### Intervention

According to the classification by Wiener et al. [16], tight control is divided into two categories based on target glucose levels: very tight control (< 110 mg/dL) and moderately tight control (< 150 mg/dL). Additionally, considering the classification of loose control from other studies^[11–13,17−25]^, it can be broadly divided into two categories: loose control (< 180 mg/dL) and very loose control (< 252 mg/dL). Therefore, in our study, we categorized blood glucose control strategies into four groups: strict control (< 110 mg/dL), intermediate strict control (< 150 mg/dL), liberal control (< 180 mg/dL), and very liberal control (< 252 mg/dL).

### Outcome

Considering that the data included in our study primarily come from subgroup analyses of randomized controlled trials (RCTs), the available data are limited. Therefore, the primary outcome of our study is all-cause 90-day mortality. For studies where 90-day mortality was not reported, we used the closest available time point to day 90.

### Statistically analysis

To compare the efficacy of each glucose control strategy in reducing all-cause mortality, we conducted a network meta-analysis across all studies. The analysis was performed using the “mvmeta” package in Stata 17. Risk ratios with 95% confidence intervals (CIs) were used to present the combined outcomes. Based on the results of the inconsistency test, if no significant inconsistency was detected (*p* > 0.05), a consistency model was applied; otherwise, an inconsistency model was used. The analysis was performed using a random effects model. We plotted cumulative probability curves for all interventions. In cumulative probability curves, the x-coordinate of each intervention represents the ranking, while the y-coordinate represents the probability of that ranking. The area under the cumulative probability curve for each intervention is referred to as the surface under the cumulative ranking (SUCRA). SUCRA is a metric used to rank treatments based on their effectiveness in a network meta-analysis (NMA). It calculates the probability that a particular intervention will perform better than the other interventions included in the analysis. SUCRA values range from 0 to 1, where 1 represents the best possible treatment and 0 represents the worst. A higher SUCRA value indicates a greater likelihood of being the most effective treatment, while a lower value suggests a lower probability of achieving the best outcome. We plotted a funnel plot to subjectively assess small-study effects in publication bias. Small-study effects refer to the tendency of smaller studies to have greater variability and potentially report exaggerated effect sizes. If the distribution of studies in the funnel plot is asymmetric (e.g., smaller studies favor positive results), it may indicate small-study effects. Additionally, we conducted Egger’s test to objectively evaluate small-study effects. If the *p*-value of Egger’s test is greater than 0.05, there is no clear evidence of small-study effects. We conducted sensitivity analysis using the node-splitting method and loop consistency test to explore inconsistencies between studies. The node-splitting method is a technique in network meta-analysis used to detect inconsistency. It separates direct and indirect evidence for each intervention pair and compares them to assess consistency. A significant difference between the direct and indirect evidence suggests potential inconsistency in the network, which may require further adjustment. This method helps ensure the reliability and accuracy of the analysis. The loop consistency test is used to assess the consistency of indirect comparisons between treatments in network meta-analysis. By examining the consistency of loops formed by treatments, it identifies potential inconsistencies in the network. A significant difference between direct and indirect evidence within a loop indicates possible inconsistency, which may require further adjustments. This test also contributes to ensuring the reliability and accuracy of the results. A *p*-value < 0.05 is considered to indicate a statistically significant difference in this study.

## Result

### Study selection

**Fig. 1:**
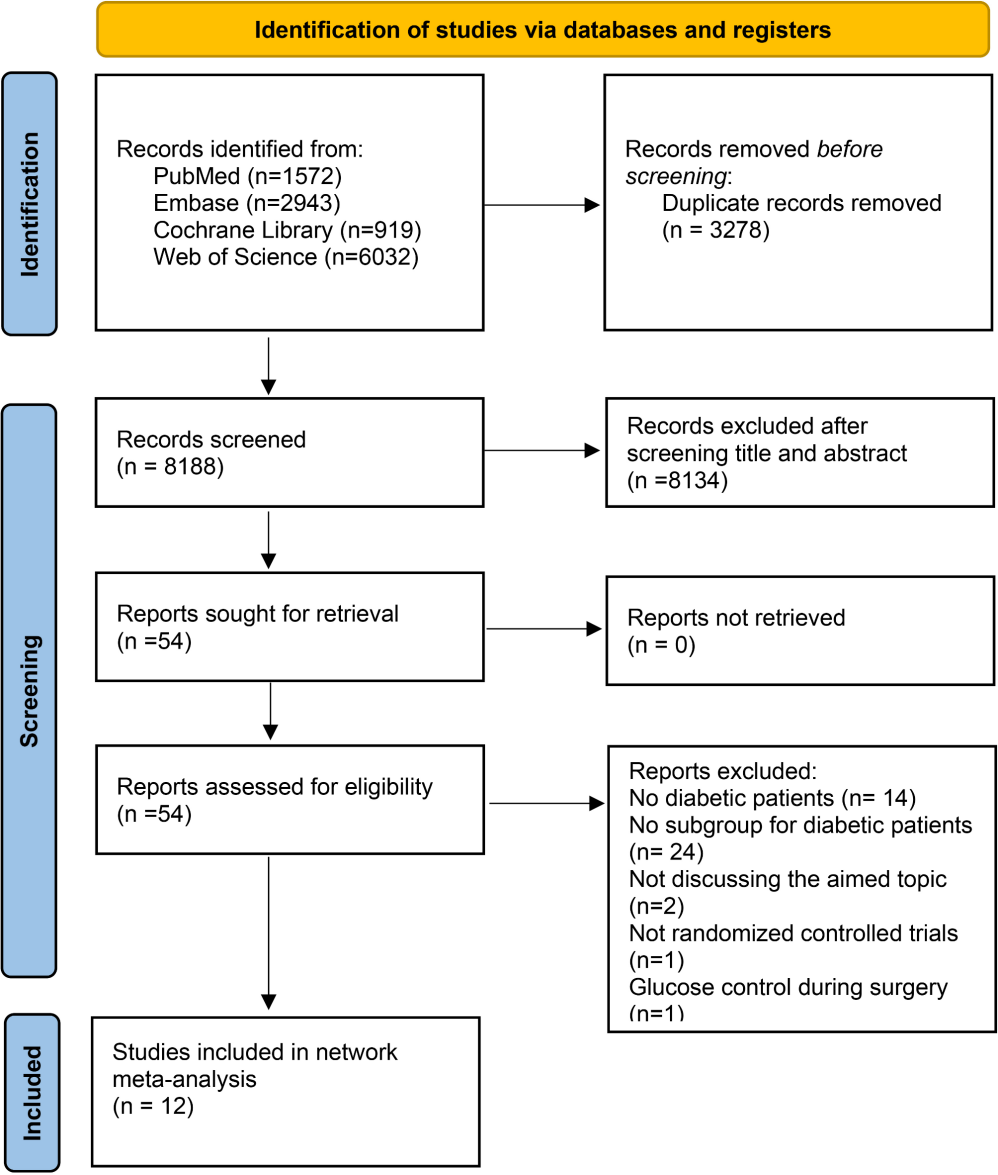
Flowchart of study selection

The database search identified 11,466 records: 1,572 from PubMed, 2,943 from Embase, 919 from the Cochrane Library, and 6,032 from Web of Science. After removing 3,278 duplicates, we screened the titles and abstracts of 8,188 records. A total of 8,134 records were excluded. Following a full-text review of the remaining 54 articles, we ultimately included 12 randomized controlled trials^[11–13,17−25]^ in this network meta-analysis (Fig. 1).

### Study characteristic and risk of bias

The included studies primarily focused on adult patients, although the reasons for ICU admission and patient types varied. These studies included medical and surgical patients (6 studies), medical-only patients (1 study), surgical-only patients (4 studies), and sepsis (1 study). Among the four studies focusing on surgical-only patients, two involved cardiac surgery, and one involved gastrectomy. Of the included studies, nine focused on both type 1 and type 2 diabetes, while three specifically targeted type 2 diabetes. Six studies had a follow-up duration of 90 days, while six studies had a follow-up duration of either during hospitalization or 28 days (Table 1). It should be noted that in Table 1, due to limited data from diabetic patients, the achieved glucose levels were observed values from the total population. The glucose control protocols, accuracy of the glucose measurements, and feeding strategies for the included studies were presented in eTable1 in supplement.

**Table 1:** Characteristic of included studies

Registration Number	Study	Country	Category of ICU	Participants Number		Glucose control in intervention group		Glucose control in control group		Type of diabetes mellitus	Definition of Severe hypoglycemia (mg/dL)	Follow-up time
				Intervention	Control	Target glucose (mg/dL)	Classification	Target glucose (mg/dL)	Classification			
ISRCTN07413772	Arabi et al. (2008) [25]	Saudi Arabia	Medical and surgical	85	123	80–110	Strict control	180–200	Very liberal control	Type2	40	During hospital
NA	Van den Berghe et al. (2001) [24]	Belgium	Surgical	101	103	80–110	Strict control	180–200	Very liberal control	Type1 and Type2	40	During hospital
NCT00115479	Van den Berghe et al. (2006) [23]	Belgium	Medical	106	97	80–110	Strict control	180–200	Very liberal control	Type1 and Type2	40	90d
NCT00135473	Brunkhorst et al. (2008) [22]	German	Sepsis	72	91	80–110	Strict control	180–200	Very liberal control	Type1 and Type2	40	28d
NA	Cao et al. (2011) [21]	China	Gastrectomy	92	87	80–110	Strict control	180–200	Very liberal control	Type2	40	28d
NCT00220987	Finfer et al. (2009) [20]	Austria and New Zealand	Medical and surgical	615	596	81–108	Strict control	144–180	Liberal control	Type1 and Type2	40	90d
NCT03665207	Gunst et al. (2023) [19]	Belgium	Medical and surgical	933	955	80–110	Strict control	180–215	Very liberal control	Type1 and Type2	40	90d
NCT01002482	Kalfon et al. (2014) [18]	France	Medical and surgical	262	274	80–110	Strict control	< 180	Liberal control	Type1 and Type2	40	90d
NA	De la Rosa et al. (2008) [17]	Colombia	Medical and surgical	32	29	80–110	Strict control	180–200	Very liberal control	Type1 and Type2	40	During hospital
NCT00460499	Lazar et al. (2011) [11]	USA	CABG	209	210	90–120	Intermediate strict control	120–180	1 Liberal control	Type1 and Type2	80	During hospital
ACTRN 12,616,001,135,404	Poole et al. (2022) [12]	New Zealand	Medical and surgical	74	76	108–180	Liberal control	< 252	Very liberal control	Type2	54	90d
NCT01361594	Umpierrez et al. (2015) [13]	USA	CABG	38	37	100–140	Intermediate strict control	141–180	Liberal control	Type1 and Type2	40	90d

CABG: Coronary Artery Bypass Grafting; d, day; NA, not available

**Fig. 2:**
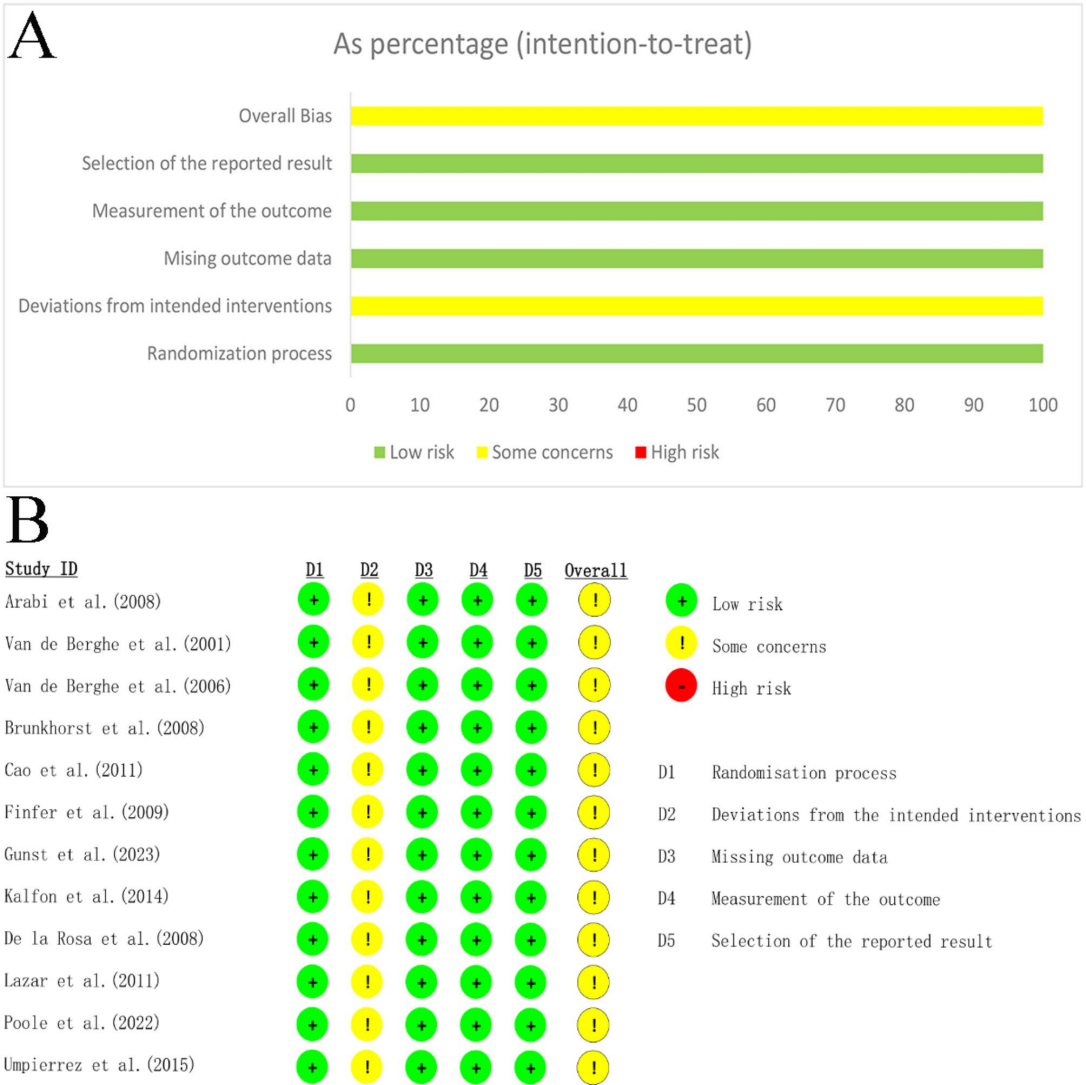
Overall distribution of risk of bias (**A**) and risk of bias of each included studies (**B**)

In the 12 studies, all raised some concerns about bias. The main source of bias in most studies was the inability to implement blinding for patients (Fig. 2). It is also important to note that, represented by the average achieved glucose level, only three studies among the 12 included achieved the target glucose level. However, in the group that did not reach the target, the achieved glucose level in the strict group was much lower than in the liberal group.

### Network meta-analysis for all-cause mortality

**Fig. 3:**
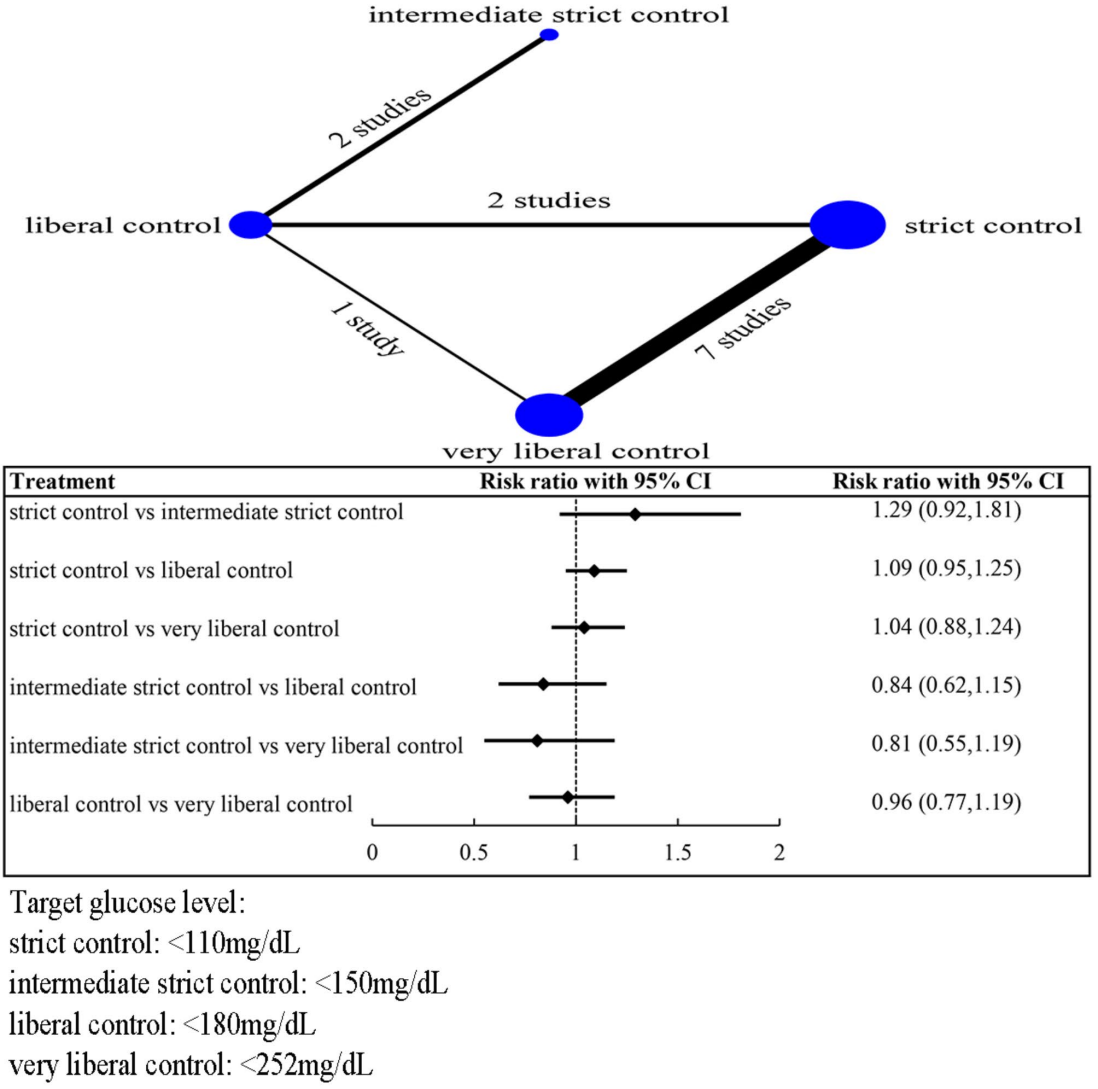
Network plot and network meta-analysis between strict control, intermediate strict control, liberal control, very liberal control. (Network plot: The size of each circle reflects the number of participants included, and the thickness of the lines between the circles indicates the number of studies comparing the various strategies. Forest plot: When the RR value is greater than 1, it indicates that the former (more strict control group) has a higher mortality rate compared to the latter (less strict control group). Conversely, when the RR value is less than 1, it means the former has a lower mortality rate. Each diamond represents the pooled RR of direct and/or indirect comparisons for each study, with the lines at both ends representing the 95% confidence interval. The dashed line crossing one represents the null line, and any result crossing one indicates no statistically significant difference.)

**Fig. 4:**
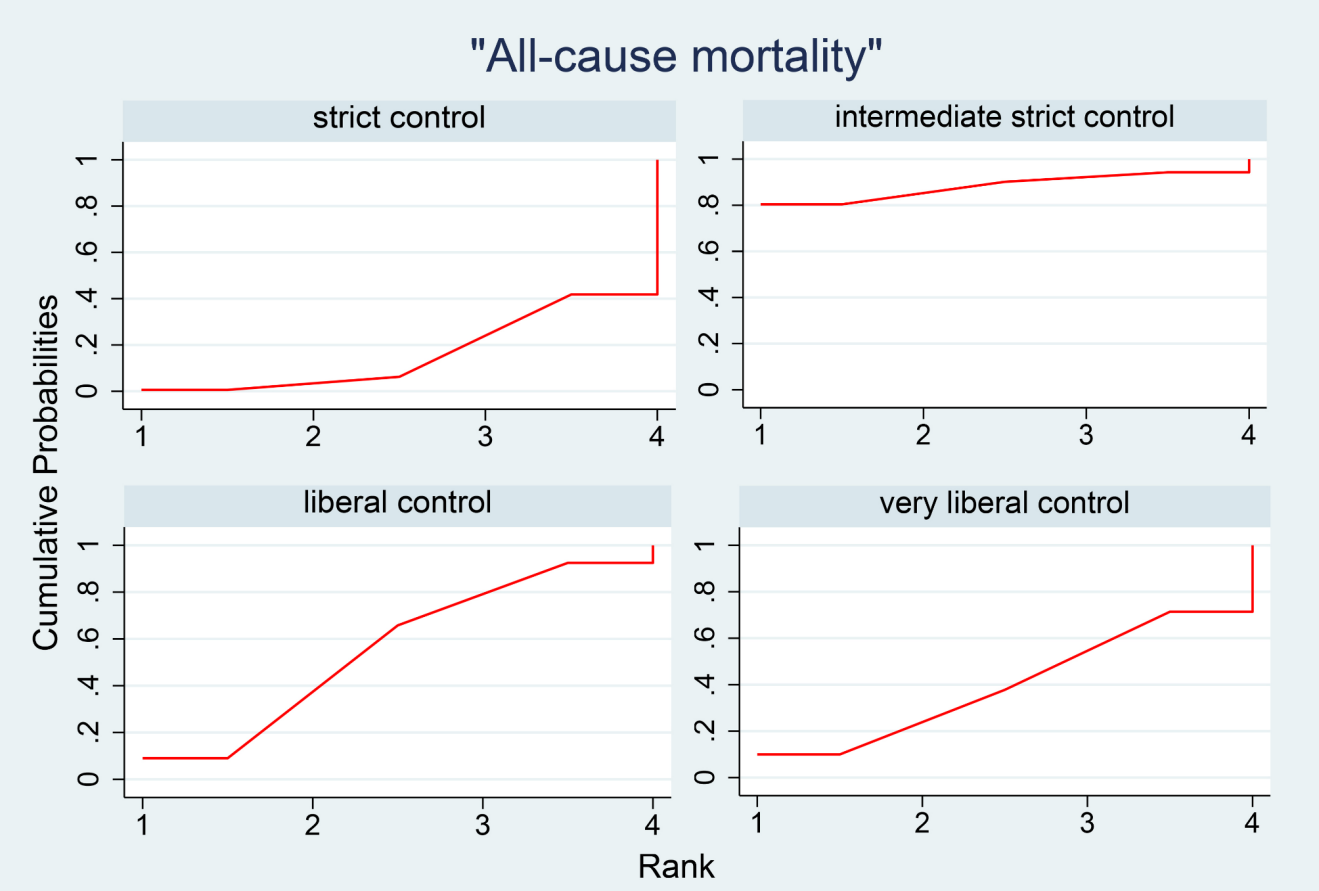
Cumulative probability of the ranking of strict control, intermediate strict control, liberal control and very liberal control (Each line represents a control strategy, and each line has four data points. The x-coordinate of each data point represents the ranking, while the y-coordinate represents the probability of that ranking.)

**Fig. 5:**
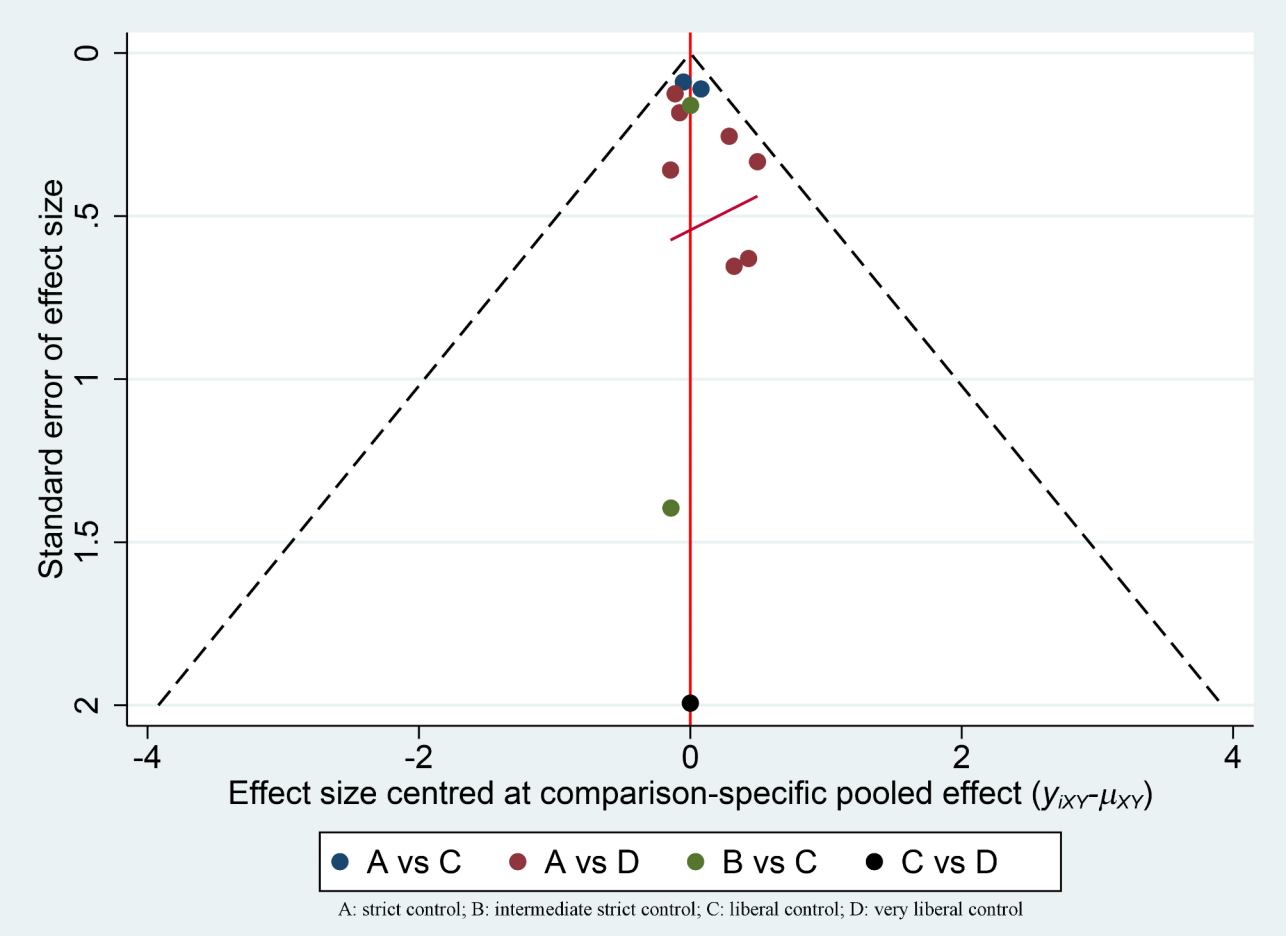
Funnel plot of included studies (If the funnel plot is relatively symmetrical, it suggests a lower likelihood of publication bias. If the funnel plot is asymmetrical, particularly at the bottom, it indicates a potential presence of publication bias or small sample effects. The solid red oblique line in the middle represents the result of Egger’s test a slope of 0.16 (95% CI, 0.15 to 0.18; *p* = 0.542 > 0.05))

Twelve studies assessed the all-cause mortality of various glucose control strategies in ICU patients with diabetes. Based on the differences in target glucose levels between the intervention and control groups in each study, we classified the studies according to the classification method described in the Methods section (Table 1). Figure 3 shows the distribution of comparisons between the strategies, where the size of the circles reflects the number of participants included in each strategy, and the thickness of the lines connecting the strategies indicates the number of studies. We observed that the strict control group and very liberal control group had the largest number of participants, and the most studies compared these two strategies. Inconsistency testing revealed a *P*-value of 0.9731, which is greater than 0.05, indicating no significant inconsistency. Therefore, we used a consistency model for the analysis. The final network meta-analysis results showed no significant statistical differences between the four glucose control strategies. At the same time, we have also presented the comparison results of the various blood glucose control strategies in the Supplement with league diagram (eTable2). The contribution distribution of the various studies in this network meta-analysis was showed in eFigure1. To further compare the effectiveness of these four strategies in controlling all-cause 90-day mortality, we plotted the cumulative probability for each strategy’s ranking, which shows the relative positions of each strategy. Each line represents a control strategy, and each line has four data points. The x-coordinate of each data point represents the ranking, while the y-coordinate represents the probability of that ranking. For example, the four points for intermediate strict control are (1, 0.799), (2, 0.901), (3, 0.94), and (4, 1), which indicate that the probability of liberal control being ranked first is 0.799, the probability of being in the top two is 0.901, the probability of being in the top three is 0.94, and the probability of being in the top four is 1 (Fig. 4). Using the cumulative probability curves, we calculated the area under the curve (SUCRA) for each strategy. The strategies, ranked from highest to lowest SUCRA, were as follows: intermediate strict control (SUCRA, 88%), liberal control (SUCRA, 55.3%), very liberal control (SUCRA, 40.3%), and strict control (SUCRA, 16.5%). Therefore, intermediate strict control might be the most effective strategy for reducing all-cause mortality among four. The funnel plot showed that the studies were mainly concentrated at the top, with a relatively balanced distribution, suggesting no significant publication bias (Fig. 5). The solid red oblique line in the middle of Fig. 5 represents the result of Egger’s test. The Egger’s test analysis shows a slope of 0.16 (95% CI, 0.15 to 0.18; *p* = 0.542 > 0.05), indicating no significant evidence of small sample study effects (eFigure1). As there was no direct comparison between intermediate strict control and very liberal control, and no direct comparison between strict control and intermediate strict control, the node-splitting method only analyzed results with both direct and indirect evidence. The findings revealed that the comparisons of strict control vs. liberal control, strict control vs. very liberal control, intermediate strict control vs. liberal control, and liberal control vs. very liberal control all yielded *P*-values greater than 0.05 (eFigure3). Similarly, the consistency test examined the loop of strict control-liberal control-very liberal control showed *P*-values greater than 0.05 (eFigure4). Therefore, both the node-splitting method and the consistency test revealed no significant inconsistencies.

## Discussion

Our network meta-analysis is the first to compare the effects of strict control, intermediate strict control, liberal control, and very liberal control in ICU patients with diabetes. Although the SUCRA rankings suggest that intermediate strict control may have a higher priority, no significant statistical differences were observed among the four strategies in reducing all-cause mortality. One notable point in this study is that we ranked different glucose control strategies based on SUCRA. However, we acknowledge that the results from SUCRA are not entirely consistent with findings from some original randomized controlled trials (RCTs). For example, although SUCRA analysis suggests that very liberal glucose control is the third best strategy (after liberal glucose control), the only RCT comparing very liberal versus liberal glucose control found a 5% higher mortality in the very liberal control group, and the study was stopped prematurely. While this difference did not reach statistical significance in the LUCID RCT [12], the potential mortality effect was larger than what was observed in RCTs comparing very liberal versus strict control. Given the hypothesis-generating nature of these findings, we believe that the current evidence is insufficient to conclude that liberal glucose control is superior with regard to mortality. Therefore, we suggest not relying solely on the SUCRA ranking and emphasize the need for further validation of the true impact of different glucose control strategies on mortality.

In 2024, Defante et al.‘s traditional meta-analysis [8] compared strict glucose control with liberal glucose control in reducing mortality among ICU patients with diabetes. Their meta-analysis included eight RCTs and categorized blood glucose control strategies into two groups: strict control and liberal control. The target glucose level for the strict control group was 80–110 mg/dL, while the liberal control group was further divided into three subgroups: <180 mg/dL, 180–215 mg/dL, and 180–200 mg/dL. Therefore, liberal blood glucose control strategies, which involve multiple ranges, were grouped into a single category. The analysis found no statistically significant difference in all-cause mortality between strict and liberal blood glucose control in ICU patients with diabetes. Although our results also showed no statistically significant difference in all-cause mortality between each strategy, our network meta-analysis, with a comprehensive comparison and SUCRA estimates, indicated that intermediate strict control (< 150 mg/L) might be more effective than other strategies in reducing all-cause mortality.

Hypoglycemia is another issue to be considered in glucose control strategies. Several studies and meta-analyses have shown that intensive insulin therapy for strict glucose control is associated with an increased risk of hypoglycemia, which is linked to higher morbidity and mortality in hospitalized patients [26–28]. The 2024 Society of Critical Care Medicine (SCCM) guidelines recommend against targeting lower blood glucose ranges (80–139 mg/dL) due to the increased risk of hypoglycemia, instead preferring a higher target range (140–200 mg/dL) [29].

However, our study has several limitations. Firstly, there is heterogeneity across the included studies, which may stem from variations in glucose control protocols, accuracy of glucose measurements, feeding strategies, and patient populations. While these protocols aimed to maintain glucose within target ranges, their implementation varied, potentially influencing treatment outcomes, particularly long-term results and complications. Furthermore, differences in glucose measurement methods (e.g., arterial vs. capillary samples) and monitoring frequencies could have impacted the precision of glucose monitoring and the evaluation of treatment efficacy. Feeding strategies also varied, with some studies recommending early enteral feeding while others used parenteral nutrition, potentially affecting glucose management and clinical outcomes. Additionally, the included patient populations were diverse, ranging from medical and surgical ICU patients to those post-cardiac surgery, introducing variability in disease severity and treatment responses. These factors should be considered when interpreting the results, and future research could benefit from standardizing these variables for more accurate comparisons. Secondly, there were some concerns about the risk of bias in the included studies, primarily due to the difficulty in implementing blinding. However, blinding is challenging in glucose control RCTs, as it may be unsafe to blind clinicians regarding the evolution of blood glucose levels. Lastly, we only analyzed all-cause 90-day mortality. In contemporary intensive care, mortality may be difficult to impact significantly. Morbidity outcomes (e.g., incidence of infections, recovery from organ failure) may be more sensitive to interventions. However, the data for this analysis mainly come from subgroup analyses in certain RCTs focusing on the primary outcomes for diabetic patients, limiting us to extract data on all-cause mortality. As a result, we were unable to analyze important side effects of glucose control, such as severe hypoglycemia. Due to the limited data, we could not conduct a comprehensive comparison of the four glucose control strategies in other aspects.

## Conclusion

This study included 12 randomized controlled trials that compared strict control, intermediate strict control, liberal control, and very liberal control strategies. The results showed no significant differences among the four glucose control strategies in reducing all-cause 90-day mortality in ICU patients with diabetes. Therefore, the current evidence is insufficient to conclude that any particular strategy is clearly superior to the others in reducing mortality. Further research is needed to validate these preliminary hypothesis-generating findings.

## Electronic supplementary material

Below is the link to the electronic supplementary material.

Supplementary Material 1

## Data Availability

All data generated or analyzed are included in this article.
