# Supplementary material for: Comparison of all-cause mortality with different blood glucose control strategies in patients with diabetes in the ICU: a network meta-analysis of randomized controlled trials
Source: Ann Intensive Care. 2025 Apr 9;15:51. doi: 10.1186/s13613-025-01471-x (PMC11982002; doi:10.1186/s13613-025-01471-x)
Supplement: Supplementary file 1 — Supplementary Material 1 [file 13613_2025_1471_MOESM1_ESM.docx]

**Supplement**

1. **Search strategy**
2. **eTable1. Glucose control protocols, accuracy of the glucose measurement, and feeding strategy of included studies**
3. **eTable2. League diagram of glucose control strategy on all-cause 90-day mortality**
4. **eFigure1. Results of Egger’s test**
5. **eFigure2. The contribution of different strategy in network meta-analysis**
6. **eFIgure3. Results of node-splitting method**
7. **eFigure4. Results of loop consistency test**

**Search strategy**

**PubMed**

1. "Intensive Care Units"[Mesh] OR "Intensive Care Units"[tiab] OR “Intensive Care Unit”[tiab] OR “Unit, Intensive Care”[tiab] OR “ICU Intensive Care Units”[tiab] OR “ICU”[tiab] OR “critically”[tiab]
2. "Glucose"[Mesh] OR “glucose”[tiab] OR “Dextrose”[tiab]
3. “Control”[tiab]
4. #2 AND #3
5. #1 AND #4
6. ((compar*[tiab]) OR ((singl*[tiab] OR doubl*[tiab] OR tripl*[tiab]) and (mask*[tiab] OR blind*[tiab]))) OR (random*[tiab] OR placebo[tiab] OR controlled[tiab] OR trial*[tiab])
7. #5 AND #6

**Cochrane Library**

1. MeSH descriptor: [Intensive Care Units] explode all trees
2. (Intensive Care Units OR Intensive Care Units OR Intensive Care Unit OR Unit, Intensive Care OR ICU Intensive Care Units OR ICU OR critically):tiab
3. #1 OR #2
4. MeSH descriptor: [Glucose] explode all trees
5. (Glucose OR Dextrose):ti,ab
6. #4 OR #5
7. (control):ti,ab
8. #6 AND #7
9. #3 AND #8
10. ((compar*) OR ((singl* or doubl* or tripl*) and (mask* or blind*))) OR (random* or placebo or controlled or trial*):ti,ab
11. #9 AND #10

**Embase**

1. 'intensive care unit'/exp
2. ((Intensive Care Units) OR (Intensive Care Units) OR (Intensive Care Unit) OR (Unit, Intensive Care) OR (ICU Intensive Care Units) OR (ICU) OR (critically)):ti,ab
3. #1 OR #2
4. 'glucose'/exp
5. ((glucose) OR (Dextrose)):ti,ab
6. #4 OR #5
7. (control):ti,ab
8. #6 AND #7
9. #3 AND #8
10. compar* OR ((singl* OR doubl* OR tripl*) AND (mask* OR blind*)) OR random*:ti,ab OR placebo:ti,ab OR controlled:ti,ab OR trial*:ti,ab
11. #9 AND #10

**WOS**

1. TS=(Intensive Care Units OR Intensive Care Units OR Intensive Care Unit OR Unit, Intensive Care OR ICU Intensive Care Units OR ICU OR critically)
2. TS=(Glucose OR Dextrose)
3. TS=(Control)
4. #2 AND #3
5. #1 AND #4
6. TS=(((compar*) OR ((singl* or doubl* or tripl*) and (mask* or blind*))) OR (random* or placebo or controlled or trial*))
7. #5 AND #6

eTable1.

| **Study** | **Glucose control protocols** | **Accuracy of the glucose measurement** | **Feeding strategy** |
| --- | --- | --- | --- |
| Arabi et al (2008) | Insulin (250 units of Hu mulin R, Eli Lilly and Company, Indianapolis, IN) mixed in 250 mL of 0.9% normal saline was infused using a volumetric infusion pump. Insulin protocols were implemented by the bedside nurses. In the IIT group, insulin infusion was adjusted to maintain a blood glucose level of 4.4 to 6.1 mmol/L (80–110 mg/dL). If the blood glucose levels fell below 4.4 mmol/L (80 mg/dL), insulin infusion was reduced or stopped. In the CIT, insulin infusion was adjusted to maintain a blood glucose level of 10.0–11.1 mmol/L (180–200 mg/dL). If the blood glucose level fell below 10.0 mmol/L (180 mg/dL), insulin was reduced or stopped. | Blood glucose was checked hourly using arterial or capillary whole blood samples using a glucose analyzer. Frequency of blood glucose monitoring increased to every 20 mins when blood glucose levels decreased to 3.2 mmol/L (58 mg/dL) and reduced to every 2–4 hours when measurements were stable. | Early in the ICU course, enteral feeding being the standard route. Caloric requirement was estimated by a dietitian using the Harris-Benedict equations and adjusting for stress factors. Protein requirement was calculated as 0.8–1.5 g/kg based on the patient condition and underlying diseases. |
| Van den Berghe et al (2001) | In the conventional-treatment group, a continuous infusion of insulin in 50 ml of 0.9 percent sodium chloride), with the use of a pump, was started only if the blood glucose level exceeded 215 mg per deciliter, 8,9 and the infusion was adjusted to maintain the level at a value between 180 and 200 mg per deciliter (10.0 and 11.1 mmol per liter).  In the intensive-treatment group, an insulin infusion was started if the blood glucose level exceeded 110 mg per deciliter, and the infusion was adjusted to maintain normoglycemia (80 to 110 mg per deciliter [4.4 to 6.1 mmol per liter]). The maximal dose of insulin was arbitrarily set at 50 IU per hour. When the patient was dis charged from the intensive care unit, a conventional approach was adopted (maintenance of blood glucose at a level between 180 and 200 mg per deciliter). | The blood glucose level was measured on admission and daily at 6 a.m., and daily maximal and minimal blood glucose levels were determined. Laboratory staff were unaware of the treatment assignments. | On admission, all patients were fed continuously with intravenous glucose (200 to 300 g per 24 hours). The next day, total parenteral, combined parenteral and enteral, or total enteral feeding was instituted according to a standardized schedule, with 20 to 30 non-protein kilocalories per kilogram of body weight per 24 hours and a balanced composition (including 0.13 to 0.26 g of nitrogen per kilogram per 24 hours and 20 to 40 percent of nonprotein calories in the form of lipids). Total enteral feeding was attempted as early as possible. |
| Van den Berghe et al (2006) | In the conventional-treatment group, continuous insulin infusion with the use of a pump, was started only when the blood glucose level exceeded 215 mg per deciliter (12 mmol per liter) and was adjusted to maintain a blood glucose level of between 180 and 200 mg per deciliter (10 and 11 mmol per liter). When the blood glucose level fell below 180 mg per deciliter, the insulin infusion was tapered and eventually stopped.  In the intensive-treatment group, insulin in fusion was started when the blood glucose level exceeded 110 mg per deciliter (6.1 mmol per liter) and was adjusted to maintain normoglycemia (80 to 110 mg per deciliter [4.4 to 6.1 mmol per liter]). The maximal continuous intravenous insulin infusion was arbitrarily set at 50 IU per hour. At the patient’s discharge from intensive care, a conventional approach was adopted (maintenance of blood glucose at 200 mg per deciliter or less). | Blood glucose levels that were measured on admission and daily in the morning during the study, and hypoglycemic events (defined as blood glucose levels of ≤40 mg per deciliter [2.2 mmol per liter]) were analyzed. | When patients were hemodynamically stable, enteral feeding was started according to routine guidelines. The guidelines aimed at a total of 22 to 30 kcal per kilogram of body weight per 24 hours with balanced composition (0.08 to 0.25 g of nitrogen per kilogram of body weight per 24 hours and 20 to 40 percent of nonprotein kilo calories as lipids). Enteral feeding was attempted as early as possible. |
| Brunkhorst et al (2008) | In the conventional-therapy group, a continuous insulin infusion in 50 ml of 0.9% saline solution was delivered through a perfusion pump when the blood glucose level exceeded 200 mg per deciliter (11.1 mmol per liter); the insulin level was then adjusted to maintain a blood glucose level of 180 mg per deciliter (10.0 mmol per liter) to 200 mg per deciliter. In the intensive-therapy group, infusion of insulin was started when blood glucose levels exceeded 110 mg per deciliter; the insulin level was then adjusted to maintain euglycemia (80 to 110 mg per deciliter). The insulin dose was adjusted to whole-blood glucose levels, which were measured at intervals of 1 to 4 hours with the use of either arterial or capillary blood samples and a glucometer. ICU nurses calculated insulin adjustments with the use of the Leuven titration guidelines. | Whole-blood glucose levels were measured at intervals of 1 to 4 hours with the use of either arterial or capillary blood samples and a glucometer | Not available |
| Cao et al (2011) | In IG treatment, the insulin infusion was started if the blood glucose levels exceeded 6.1 mmol/l and was adjusted to maintain the blood glucose target between 4.4 and 6.1 mmol/l. In CG treatment, the insulin infusion was started if the blood glucose level exceeded 12.0 mmol/l and was adjusted to maintain the blood glucose target between 10.0 and 11.0 mmol/l. | Blood glucose levels were measured and monitored using a bedside glucometer or laboratory analyzers in routine use. The glucometers were calibrated regularly by the manufacturer to ensure the accuracy and reliability. | Not available |
| Finfer et al (2009) | The intensive (i.e., tight) control target of 81 to 108 mg per deciliter (4.5 to 6.0 mmol per liter), based on that used in previous studies,12,13 or a conventional-control target of 180 mg or less per deciliter (10.0 mmol or less per liter).  Control of blood glucose was achieved with the use of an intravenous infusion of insulin in saline. In the group of patients assigned to undergo conventional glucose control, insulin was administered if the blood glucose level exceeded 180 mg per deciliter; insulin administration was reduced and then discontinued if the blood glucose level dropped below 144 mg per deciliter (8.0 mmol per liter). | Blood samples for glucose measurement were obtained by means of arterial catheters whenever possible; the use of capillary samples was discouraged. Blood glucose levels were measured with the use of point-of-care or arterial blood gas analyzers or laboratory analyzers in routine use at each center. | Not available |
| Gunst et al (2023) | In the patients assigned to tight glucose control, the blood-glucose level was targeted at 80 to 110 mg per deciliter (4.4 to 6.1 mmol per liter). The insulin doses, glucose doses in case of hypoglycemia, and frequency of blood-glucose measurement (ranging from every 1 to 4 hours and more frequently after hypoglycemia) were adjusted in accordance with the computer algorithm.  In the patients assigned to liberal glucose control, insulin was initiated only when the blood-glucose level exceeded 215 mg per deciliter (>11.9 mmol per liter) on two consecutive measurements (or one measurement in patients with type 1 diabetes), after which bedside physicians and nurses adjusted the insulin dose to a target blood-glucose level between 180 and 215 mg per deciliter (10.0 and 11.9 mmol per liter). | Blood-glucose levels were measured in arterial blood with use of a blood gas analyzer, and insulin was administered only as a continuous intravenous infusion through a central venous catheter. When the arterial catheter was removed, capillary blood could be used for blood-glucose measurements. | All the patients received enteral nutrition as soon as possible. When enteral nutrition was in sufficient to meet the caloric target, parenteral nutrition was initiated only after 1 week in the ICU. In all patients who did not receive 80% of the nutritional intake enterally, parenteral micro nutrients were administered according to local practice in order to prevent refeeding syndrome. |
| Kalfon et al (2014) | Tight computerized glucose control was performed with the assistance of the CGAO software set for targeting a low BG range of 4.4–6.1 mmol/L (80–110 mg/dL).  Glucose control was based on current practice already used in the participating ICU before the beginning of the study, and the target BG was B10.0 mmol/L (180 mg/ dL). | At least one BG value per day was measured by the hospital central laboratory on a morning sample. | Enteral feeding was attempted as early as possible according international guidelines. |
| De la Rosa et al (2008) | Both groups received insulin via continuous infusion pump. The standard concentration of insulin was 100 units in 100 ml of 0.9% saline solution. In the standard insulin group, insulin infusion was started when glucose levels exceeded 215 mg/dl and was adjusted to maintain blood glucose levels between 180 and 200 mg/dl (10.0 to 11.1 mmol/L). In the intensive insulin group, insulin infusion was started when blood glucose levels exceeded 110 mg/dL, and was adjusted to maintain a glucose level of between 80 and 110 mg/dl (4.4 to 6.1 mmol/L). | Blood glucose levels were measured in undiluted arterial blood. Undiluted samples were obtained by removing at least four times the flush-volume in the line between the sampling point and the arterial puncture site before the actual sample was taken or, when an arterial catheter was not available, in capillary blood with the use of a point-of-care glucometre. Glucose levels were determined with a glucometre at admission to ICU. They were repeated every one, two and four hours if the patient had insulin infusion, and every four and six hours if no insulin was required according to the algorithm | Not available |
| Lazar et al (2011) | Patients were randomly assigned to either a moderate group (serum glucose 120-180 mg/dL) or an aggressive group (90 120 mg/dL). After induction of general anesthesia, a continuous insulin infusion with 100 units of regular insulin in 100 mL of 0.9% normal saline was initiated at 3 mL/hour and titrated to maintain the targeted glucose level on the basis of the algorithm. | Blood glucose measurements were obtained by means of arterial catheters and were measured with the use of point-of-care analyzers. Fatty acids were measured before induction and at 0, 6, and 18 hours after admission to the ICU. | Not available |
| Poole et al (2022) | Participants assigned to liberal glucose control (the intervention group) had intravenous insulin commenced at a blood 876 glucose .252 mg/dl and titrated to a target range of 180–252 mg/dl. If the blood glucose was ,180 mg/dl, no attempt to increase blood glucose was made, with the exception of local protocols for management of hypoglycemia. Participants assigned to usual care (comparator group) had intravenous insulin commenced and titrated as per preexisting protocols for the institution. | Blood glucose concentrations on Days 1–7 that were recorded as part of routine clinical care nearest to the trial time points of 06:00, 12:00, 18:00, and 24:00 were recorded as trial blood glucose values. If no sample was taken as part of routine clinical care within 3 hours of the designated interval, this time point was recorded as missing. If the daily minimum or maximum blood glucose concentrations obtained as part of routine clinical care occurred outside of the set trial time points (06:00, 12:00, 18:00, and 24:00), these values were recorded separately. On study Days 8–14, the blood glucose closest to 08:00 was recorded. The choice of arterial, venous, or capillary blood and testing technique was at the discretion of the treating clinician. | Not available |
| Umpierrez et al (2015) | Patients assigned to the intensive group received continuous insulin infu sion (CII) adjusted to maintain a glucose target between 100 and 140 mg/dL in the ICU. Those assigned to conservative control received CII adjusted to maintain a glucose level between 141 and 180mg/dL in the ICU. | Not available | Not available |

eTable2. League diagram of glucose control strategy on all-cause 90-day mortality (The RR value in the upper right corner represents the intervention below compared to the intervention above. For example, 1.05 (0.88, 1.26) represents the RR value for mortality of Intermediate strict glucose control (intervention below) vs Strict glucose control (intervention above). The RR value in the lower left corner represents the intervention above compared to the intervention below. For example, 0.95 (0.80, 1.14) represents the RR value for mortality of Strict glucose control (intervention above) vs Intermediate strict glucose control (intervention below). Similarly, 0.87 (0.73, 1.04) represents the RR value for mortality of Liberal glucose control (intervention below) vs Strict glucose control (intervention above), while 1.14 (0.96, 1.36) represents the RR value for mortality of Strict glucose control (intervention above) vs Liberal glucose control (intervention below). When the RR value is greater than 1, it indicates that the former has a higher mortality rate compared to the latter. Conversely, when the RR value is less than 1, it means the former has a lower mortality rate.)

| Strict glucose control | 0.78 (0.55,1.09) | 0.92 (0.80,1.05) | 0.96 (0.81,1.14) |
| --- | --- | --- | --- |
| 1.29 (0.92,1.81) | Intermediate strict glucose control | 1.18 (0.87,1.62) | 1.23 (0.84,1.81) |
| 1.09 (0.95,1.25) | 0.84 (0.62,1.15) | Liberal glucose control | 1.04 (0.84,1.30) |
| 1.04 (0.88,1.24) | 0.81 (0.55,1.19) | 0.96 (0.77,1.19) | Very liberal glucose control |

eFigure1.


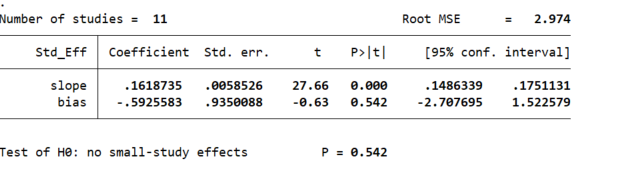


eFigure2. The contribution of different strategy in network meta-analysis


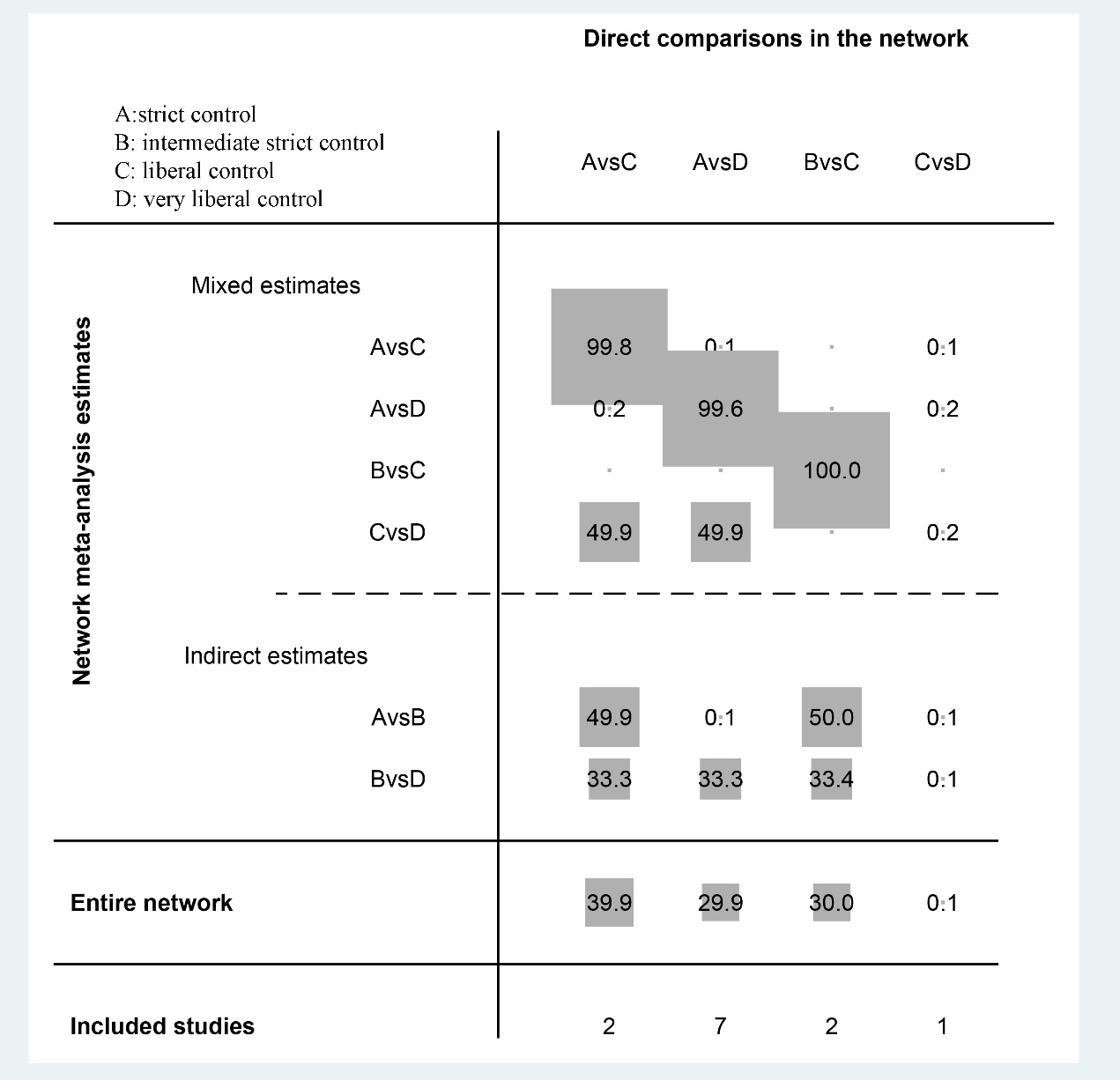


This figure shows the contribution of different glucose control strategies in a network meta-analysis. The primary aim is to illustrate the results from direct and indirect comparisons between the strategies and their contributions to the overall network analysis.

1. **Mixed Estimates (Direct Comparisons)**:
   - The "Mixed estimates" section shows the direct comparisons between the different glucose control strategies. These estimates are derived from the pairwise comparisons of strategies such as **Strict control (A) vs. Intermediate strict control (B)**, **Strict control (A) vs. Liberal control (C)**, and so on.
   - For example, the value **99.2** under **A vs. B** means that, based on the direct comparison, the contribution of the comparison between strict control and intermediate strict control is **99.2%** to the network estimate. This suggests a very strong contribution of this direct comparison to the overall results.
   - Similarly, for **A vs. C**, the value is **0.4**, indicating that this direct comparison has a much smaller contribution to the network estimate compared to other comparisons.
2. **Indirect Estimates**:
   - The "Indirect estimates" section shows comparisons that are not made directly between two strategies but rather inferred through other strategies in the network. For example, **BvsD** is an indirect estimate. Instead of directly comparing Intermediate strict control (B) with Very liberal control (D), the comparison is made by using other strategies (such as A and C) in the network.
   - The indirect estimates are generally less reliable than direct comparisons, as they involve more assumptions and could introduce higher uncertainty.
3. **Entire Network Estimates**:
   - The "Entire network" row shows the overall contribution of all the strategies when all the comparisons in the network are taken into account. This reflects the combined evidence from all strategies and comparisons, providing a global estimate for the network meta-analysis.
   - For example, for the **entire network**, the estimates for **A**, **B**, **C**, and **D** are all about **33.2%**, indicating that the contribution of each strategy to the overall result is approximately equal when considering all comparisons in the network.
4. **Study Count**:
   - The “Included studies” row indicates the number of studies that contributed to each comparison. For instance, **2 studies** contributed to **A vs. B**, and **1 study** contributed to **B vs. D**. A higher number of studies typically adds to the reliability of a comparison, while a lower number of studies may reduce the robustness of the comparison.
   - It’s important to consider the study count when interpreting the results, as a smaller number of studies may result in more variability or uncertainty in the estimates.

eFigure3. Results of node-splitting method


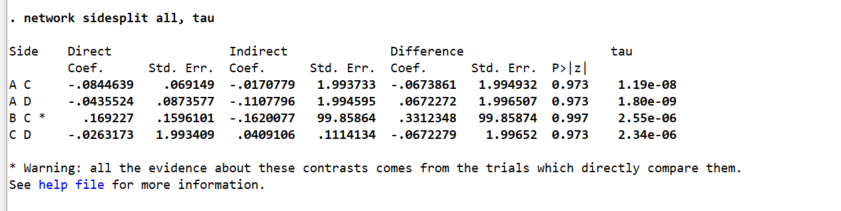


A: strict control

B: intermediate strict control

C: liberal control

D: vert liberal control

eFigure4. Results of loop consistency test


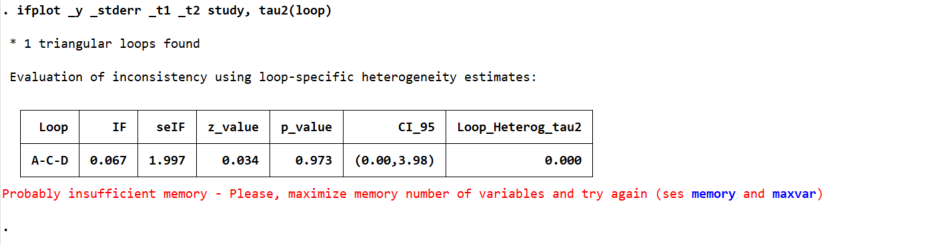


A: strict control

B: intermediate strict control

C: liberal control

D: vert liberal control
